# Development and validation of a predictive model to identify the active phase of labor

**DOI:** 10.1186/s12884-022-04946-y

**Published:** 2022-08-15

**Authors:** Simona Fumagalli, Laura Antolini, Greta Cosmai, Teresa Gramegna, Antonella Nespoli, Astrid Pedranzini, Elisabetta Colciago, Maria Grazia Valsecchi, Patrizia Vergani, Anna Locatelli

**Affiliations:** 1 School of Medicine and Surgery, University of Milano-Bicocca, Via Cadore 48, 20900 Monza, Italy; 2 Department of Obstetrics and Gynecology, MBBM Foundation at San Gerardo Hospital, Monza, Italy; 3 Department of Obstetrics and Gynecology, MBBM Foundation at San Gerardo Hospital, School of Medicine and Surgery, University of Milano-Bicocca, Monza, Italy; 4 Department of Obstetrics and Gynecology, Carate Brianza Hospital, ASST Brianza, School of Medicine and Surgery, University of Milano-Bicocca, Monza, Italy

**Keywords:** Active labor, Active phase, Midwifery diagnosis, Maternal behavior

## Abstract

**Background:**

The diagnosis of the active phase of labor is a crucial clinical decision, thus requiring an accurate assessment. This study aimed to build and to validate a predictive model, based on maternal signs and symptoms to identify a cervical dilatation ≥4 cm.

**Methods:**

A prospective study was conducted from May to September 2018 in a II Level Maternity Unit (development data), and from May to September 2019 in a I Level Maternity Unit (validation data). Women with singleton, term pregnancy, cephalic presentation and presence of contractions were consecutively enrolled during the initial assessment to diagnose the stage of labor. Women < 18 years old, with language barrier or induction of labor were excluded. A nomogram for the calculation of the predictions of cervical dilatation ≥4 cm on the ground of 11 maternal signs and symptoms was obtained from a multivariate logistic model. The predictive performance of the model was investigated by internal and external validation.

**Results:**

A total of 288 assessments were analyzed. All maternal signs and symptoms showed a significant impact on increasing the probability of cervical dilatation ≥4 cm. In the final logistic model, “Rhythm” (OR 6.26), “Duration” (OR 8.15) of contractions and “Show” (OR 4.29) confirmed their significance while, unexpectedly, “Frequency” of contractions had no impact. The area under the ROC curve in the model of the uterine activity was 0.865 (development data) and 0.927 (validation data), with an increment to 0.905 and 0.956, respectively, when adding maternal signs.

The Brier Score error in the model of the uterine activity was 0.140 (development data) and 0.097 (validation data), with a decrement to 0.121 and 0.092, respectively, when adding maternal signs.

**Conclusion:**

Our predictive model showed a good performance. The introduction of a non-invasive tool might assist midwives in the decision-making process, avoiding interventions and thus offering an evidenced-base care.

## Background

How to identify the active phase of the first stage of labor is still a matter of controversy amongst authors as well as practitioners. International guidelines are generally agreeing in characterizing it as a period of regular and painful contractions leading to a cervical dilation of 10 cms. Most of the existing divergencies appear to concern which cervical dilation represents the starting point for the labor diagnosis, and recommendations varies indeed from 4 [1] to 5 [2] to 6 cms [3, 4]. Moreover, the need for a holistic assessment [1], transcending the sole consideration of uterine activity and cervical dilatation, to identify the active phase of labor reflects the complexity of the matter.

It is of little surprise then the diagnosis of active phase of labor has been described as one of the most important yet difficult judgments to make by providers of maternity care [5–7]. An inaccurate diagnosis of active phase of labor can lead to unnecessary early hospital admission with significant impacts on birth outcomes and women satisfaction [8, 9]. This has been mostly related to the greater likelihood of unnecessary intrapartum interventions [8–13] and negative interferences with the physiological processes of childbirth and overall birth experience.

Acknowledging this complexity, international clinical guidelines recommend to offer a one-to-one and face-to-face assessment for at least 1 h prior to hospital admission [9, 3], to observe and identify signs and symptoms which are suggestive of an active phase of labor [1, 3, 4].

As above mentioned, the presence of regular uterine contractions and a cervical dilatation of 4-6 cm are the criteria commonly used to describe the active phase of labor [9]. About 80% of the studies appraised in a systematic review conducted in 2016, included the cervical dilatation criteria in the definition of latent and active labor, and just a few of them comprised other physiological signs and symptoms [6]. Evidence describes inaccuracy issues, increased risk of infection and women discomfort when recurrent vaginal examinations are performed during labor [14]. Thus, it should be highlighted the importance of the appropriate use of this method only when necessary to assess the progress during the entire labor.

Furthermore, none of the current guidelines are recommending the use of vaginal examination as the main single method to establish whether a woman is in active phase of first stage of labor. They rather indicate vaginal examination as a mean to confirm, if deemed necessary by the practitioner, what emerges as suggestive for the active phase of labor by other methods (e.g. observation of women’s behaviors, signs and symptoms) within an holistic assessment.

The latter, has been deemed to be crucial considering the complexity of the physiology underpinning the active labor [14].

Previous studies have focused on women’s behaviors and signs that may suggest labor onset, including: skin changes, body temperature, sweating, breathing, conversation, movement and posture, mood, energy, pain perceptions, presence and changes of the purple line, spontaneous rupture of membranes, vaginal discharge, cervical change over time, fetal movement and station and condition of the presenting part [15–19]. However, there is a lack of knowledge about the role that these signs play both in recognize the active phase of labor and in supporting midwives’ decision making.

Within the context of international recommendations and in order to provide a safe care, the use of a structured tool might assist midwives and caregivers in focusing on key elements using a holistic approach to diagnose active phase of labour.

Thus, this study aimed to build and to validate a predictive model to identify a cervical dilatation ≥4 cm based on maternal signs and symptoms.

## Methods

### Design

A prospective observational study was conducted, with the recruitment of women who reported the presence of uterine activity. Women were assessed performing a vaginal examination to detect the condition of the active phase of labor and patterns regarding maternal signs and symptoms were collected. The active phase of labor was defined as cervical dilatation ≥4 cm according to NICE guideline adopted in the research site. Each woman could receive one or more assessments. The total number of assessments were considered for the analysis.

### Setting

The research site where the data to develop the prediction model were collected, was an Obstetric Unit of a large maternity hospital in Northern Italy with approximately 2500 births/year. The Obstetric Unit hosts both low and high-risk women and offers one-to-one midwifery care throughout labor and birth to all women. According to the local protocol hospital admission and transfer to the birth suite occurs in active labor, in line with the NICE guideline [1]. The initial assessment was performed by a midwife, who evaluated maternal and fetal wellbeing. Women identified as not yet in active labor were encouraged to return home.

The study setting where data were collected to validate the prediction model was an Obstetric Unit of a Level I maternity hospital in Northern Italy with approximately 1200 low risk births/year. The local protocols about the hospital admission and the intrapartum care are similar in both research sites. The same variables collected to develop the prediction model were obtained for the validation data set.

### Participants

Inclusion criteria for the collection of both development and validation data set were: singleton pregnancy at term, cephalic presentation and presence of uterine activity. Maternal age less than 18, language barrier or induction of labor were considered exclusion criteria.

**Fig. 1:**
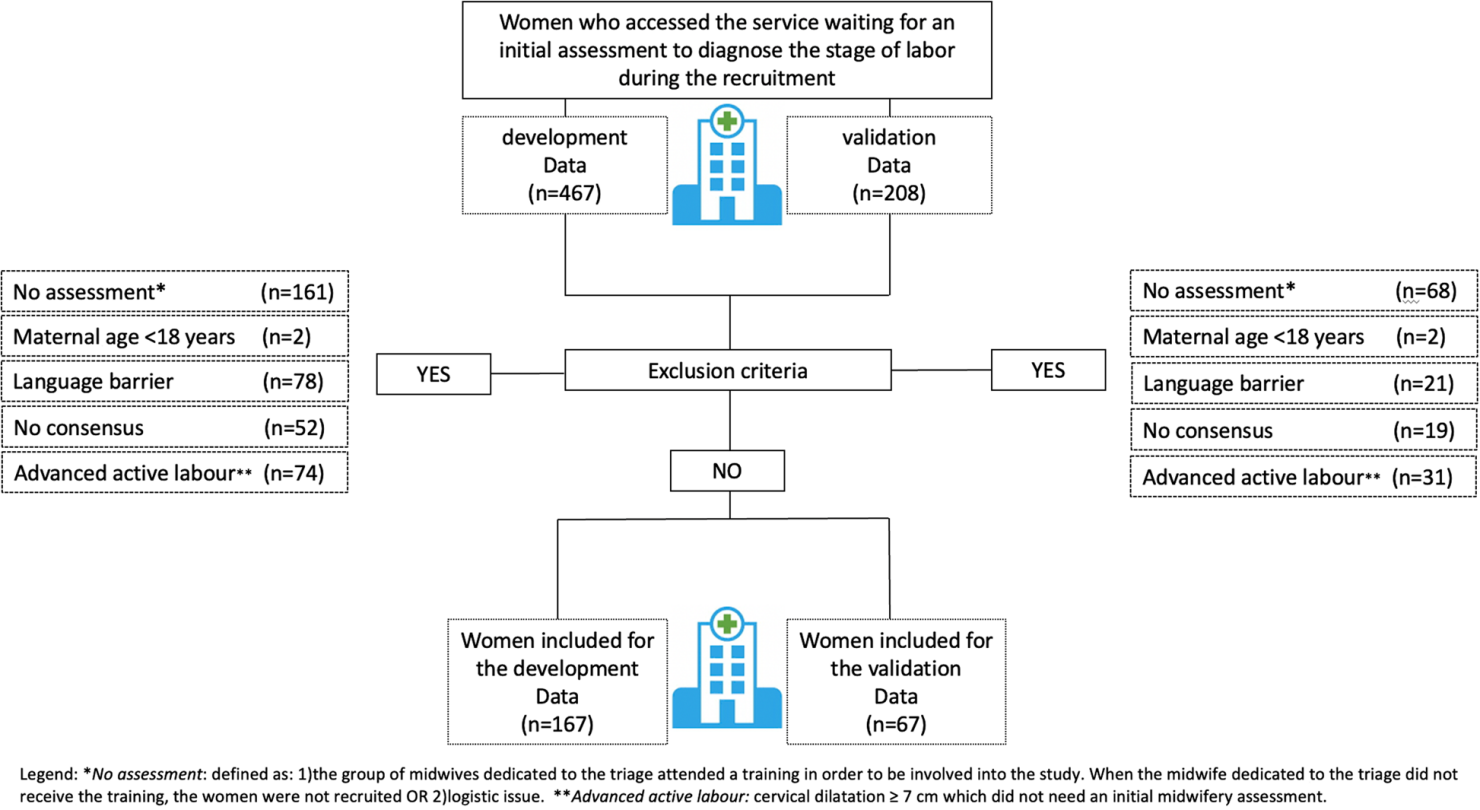
Flowchart on women involved in the study in both development and validation settings

The recruitment phase lasted 4 months (from May to September 2018 for the development data and from May to September 2019 for the validation data). Participants were enrolled consecutively at the maternity triage or at the antenatal ward to diagnose the stage of labor. In case of unavailability to perform the initial assessment (the midwife dedicated to the triage did not receive the training, OR logistic issues) the women were not recruited (Fig. 1).

Women who met the inclusion criteria were invited to take part in the study by one of the researchers, who explained the aim of the study, their involvement and asked them to sign the consent form.

### Data collection

Each assessment was performed by a midwife comprising one-to-one and face-to-face midwifery care for at least 1 hour. During this evaluation the midwife observed features of contractions (rhythm, frequency and duration) and 8 specific maternal signs. A preliminary phase of the study was conducted with the aim to define variables included into the assessment tool. A panel of 5 experienced clinical midwives identified maternal signs and symptoms significatively associated with established labor. The expert panel then defined which specific characteristics should be observed during the midwifery assessment for each of the factors identified. These data were collected as “present” or “absent” in a specific assessment tool. Overall 11 qualifiers were collected. (Table 1).

**Table 1:** Description of features of uterine activity and maternal signs included into the assessment tool

	Factors	Factors Description
Uterine activity	Frequency	More than 2 contractions in 10 minutes
	Rhythm	Regular if interval between contractions is consistent (Δ of interval < =1 minute)
	Duration	Length more then 50 seconds from the start of a contraction to its end
Maternal signs	Vaginal loss	Presence of vaginal discharge (mucousy or leukorrhea)
	Show	Presence of bloodstained mucousy vaginal discharge^a^
	Pain	Back pain referred by woman
	Breathing	Focused sigh, Vocalises, Deeper breathing
	Sweating and/or blush	Intense perspiration, Facial flushing
	Posture	Woman spontaneously adopts supportive and analgesic position. Leaning forward positions, squatting, walking, rocking and swaying^b^
	Conversation	Conversation stops, talking stops at each contraction, takes 20 seconds or more to resume talking following a contraction.
	Mood	Need of rest and/or need of physical and visual contact and/or introspective woman

^a^Marshall, Jayne E., and Maureen D. Raynor. Myles’ Textbook for Midwives E-Book, Elsevier Health Sciences, 2014. ProQuest Ebook Central, http://ebookcentral.proquest.com/lib/bournemouth-ebooks/detail.action?docID=1724272. (pag 329)

^b^Simkin P., Hanson L., Ancheta R. (2017) The Labour Progress Handbook: Early Interventions to Prevent and Treat Dystocia. John Wiley & Sons: New Jersey

Following the identification and the collection of those factors, the midwife performed a vaginal examination to evaluate the cervical dilatation. This information was dichotomized in ≥4 cm or < 4 cm.

Sociodemographic and obstetric variables were extracted from medical records.

All midwives involved in the study attended a training on the use of the assessment tool before starting the data collection.

### Statistical methods

Demographic and obstetric characteristics were described by frequency tables for categorical and discrete variables, mean and standard deviation for the continuous ones. Among categorical variables included in the initial description we considered also the number of assessments that each woman received during the study. The subsequent analysis focused on the assessment itself and not on the single woman. The percentages of presence of each feature of contractions and maternal signs were calculated according to the cervical dilatation: ≥4 cm vs < 4 cm. The relative risk of exposure was also calculated. The probability of cervical dilatation of at least 4 cm was related to each feature of contractions and maternal signs by separate logistic regression models. All factors were considered each at a time with the exception of the presence/absence of vaginal discharge that was considered jointly with the show to be a feature of discharge. Confidence intervals on ORs on the probability of cervical dilatation of at least 4 cm and related *P*-values were reported. The probability of cervical dilatation of at least 4 cm was related to features of contractions and maternal signs by three multivariate logistic regression models: 1) “Uterine activity model” (features of contractions), 2) “Maternal signs model” (maternal signs), 3) “Final model” (features of contractions and maternal signs). A nomogram for the calculation of the predictions using the Final model was also derived. This enables to predict the probability of cervical dilatation ≥4 cm has a function of features of the woman (contractions and maternal signs) assessed at any given time point, which has indeed an impact on the features. The predictive performance of each model was investigated by a descriptive analysis on the predicted probability (prediction) through the calculation of mean, standard deviation, interquartile range and range of the predictions and Brier score measure of predictive inaccuracy. The degree of separation between the predictions within the two subgroups of assessments with cervical dilatation ≥4 cm vs < 4 cm was investigated by the boxplot of the predictions and by the ROC curve. The analysis of classification errors when considering cervical dilatation < 4 cm if the prediction is lower than a fixed threshold is obtained throughout three steps: 1) the calculation of the total number of assessments with a prediction lower than the threshold, and an observed cervical dilatation ≥4 cm; 2) the calculation of the total number of assessments with a prediction lower than the threshold (regardless of the cervical dilatation observed); 3) the ratio between the two numbers 1) and 2). The same approach was used to evaluate the classification errors when considering cervical dilatation ≥4 cm. The predictive performance was assessed by calculating the prediction on both the development and the validation dataset separately.

## Results

A total of 234 women were enrolled, 167 contributed to the development data set and the remaining 67 contributed to the validation data set. The descriptive analysis of sociodemographic and obstetric variables, and number of assessments for the present study are shown in Table 2.

**Table 2:** Description of women who contributed to the development and the validation data set

Variables		Development data (*n* = 167)		Validation data (*n* = 67)	
Socio-demographic		mean	SD	mean	SD
	**Maternal age** (years)	**31.62**	5.51	**31.49**	4.49
		n	%	n	%
	**Education** (undergraduate degree or more)	**72**	43.11	**32**	47.76
	**Employed**	**121**	72.46	**48**	71.64
	**Origin**^a^ (Caucasian)	**143**	85.63	**59**	88.06
Obstetric		n	%	n	%
	**Parity** (nulliparous)	**104**	62.28	**43**	64.18
		mean	SD	mean	SD
	**Gestational age** (week)	**39.48**	0.99	**39.61**	0.97
Assessment		n	%	n	%
	**Number of assessment**				
	1	**120**	71.86	**62**	92.54
	2	**45**	26.94	**5**	7.46
	3	**2**	1.20	**0**	0.00
	Total	**216**		**72**	

^a^Non Caucasian: Asian, African, Hyspanic

Each feature of contractions and each maternal sign showed a significant impact on increasing the probability of cervical dilatation ≥4 cm. (Table 3).

**Table 3:** Separate Logistic regression models with each maternal sign as regressor (*n* = 216 observations)

Variable		Group “dil_cm < 4 cm” (*n* = 132)		Group “dil_cm ≥ 4 cm” (*n* = 84)		RR	OR	(95% CI)	***P***-value
		n	%	n	%				
Uterine Acctivity	Frequency (> 2)	**47**	35.61	**70**	83.33	2.34	**9.04**	**(**4.6;17.8)	**< 0.0001**
	Rhythm (regular)	**33**	25.00	**64**	76.19	3.05	**9.6**	**(**5.07;18.2)	**< 0.0001**
	Duration(>50″)	**44**	33.33	**73**	86.90	2.61	**13.3**	**(**6.39;27.5)	**< 0.0001**
Discharge	Vaginal Loss	**54**	40.91	**63**	75.00	1.83	**2.61**	**(**1.34;5.27)	**0.005**
	Show	**17**	12.88	**37**	44.05	3.42	**3.15**	**(**1.49;6.68)	**0.003**
Pain		**72**	54.55	**64**	76.19	1.40	**2.66**	**(**1.45;4.89)	**0.002**
Breathing		**69**	52.27	**70**	83.33	1.59	**4.56**	**(**2.34;8.9)	**< 0.0001**
Sweating and/or Blush		**47**	35.61	**51**	60.71	1.70	**2.79**	**(**1.59;4.91)	**< 0.0001**
Posture		**73**	55.30	**72**	85.71	1.55	**4.85**	**(**2.4;9.77)	**< 0.0001**
Conversation		**85**	64.39	**81**	96.43	1.50	**14.9**	**(**4.47;49.9)	**< 0.0001**
Mood		**64**	48.48	**68**	80.95	1.67	**4.51**	**(**2.37;8.59)	**< 0.0001**

The multivariate regression model showed that only “rhytm” and “duration” had a significant impact on cervical dilatation ≥4 cm (Table 4). The “Maternal signs model” showed that both “show” and “conversation” had a significant impact on the probability of cervical dilatation ≥4 cm. In the “Final model” “rhythm” and “duration”, together with “show”, confirmed their significant impact” (Table 4).

**Table 4:** Multivariate analysis on predictors of dil_cm ≥ 4 (*n* = 216 observations)

Variable		OR	(95% CI)	***P***-value
Uterine Activity model	Frequency (> 2)	**2.12**	(0.85;5.26)	**0.104**
	Rhythm (regular)	**5.09**	(2.15;12.06)	**< 0.0001**
	Duration (>50″)	**9.40**	(4.20;21.04)	**< 0.0001**
Maternal signs model	Vaginal Loss	**1.84**	(0.86;3.96)	**0.118**
	Show	**3.29**	(1.38;7.82)	**0.007**
	Pain	**1.56**	(0.74;3.28)	**0.239**
	Breathing	**1.95**	(0.81;4.71)	**0.137**
	Sweating and/or Blush	**0.86**	(0.41;1.78)	**0.681**
	Posture	**1.87**	(0.79;4.41)	**0.153**
	Conversation	**5.79**	(1.46;22.93)	**0.012**
	Mood	**1.56**	(0.66;3.67)	**0.310**
Final model	Frequency (> 2)	**1.33**	(0.46;3.81)	**0.599**
	Rhythm (regular)	**6.26**	(2.28;17.13)	**< 0.0001**
	Duration (>50″)	**8.15**	(3.15;21.11)	**< 0.0001**
	Vaginal Loss	**1.60**	(0.65;3.94)	**0.305**
	Show	**4.29**	(1.47;12.50)	**0.008**
	Pain	**1.89**	(0.77;4.64)	**0.163**
	Breathing	**1.50**	(0.49;4.62)	**0.476**
	Sweating	**0.44**	(0.17;1.16)	**0.098**
	Posture	**1.09**	(0.36;3.31)	**0.877**
	Conversation	**2.86**	(0.60;13.58)	**0.187**
	Mood	**1.23**	(0.41;3.71)	**0.711**

**Fig. 2:**
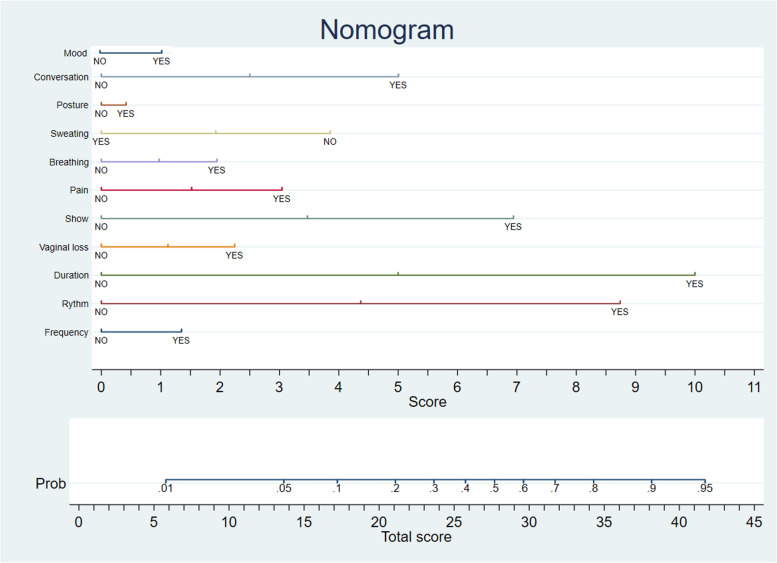
Nomogram. The predicted probability of cervical dilatation ≥4 cm is calculated in three steps: 1) the score of each signs is obtained by the vertical projection (from “NO”, “YES”) to the score axis, 2) the total score is then calculated by summing up the single score values, 3) probability of need of of cervical dilatation ≥4 cm is calculated by the vertical projection of the total score value to the to the probability of cervical dilatation ≥4 cm

The nomogram calculating the predictions using the latter model is displayed in Fig. 2.

Table 5 shows the predictive performance descriptive analysis. The standard deviation of predictions is greater in the final model compared to the other two models, showing an increment in the spread of predictions in both the development and the validation data. The Brier score inaccuracy measure is reduced when moving from the model of maternal signs to the one of features of contractions. A further reduction of the Brier score is observed in the final model. This behavior is found in both the development and the validation data. The Brier score had a better performance using the validation data.

**Table 5:** Predictive performance descriptive analysis and indicators (dil_cm ≥4)

Regression model	Development data (*n* = 216)						Validation data (*n* = 72)					
	Observed proportion of dil_cm ≥ 4	Predictive probability of dil_cm ≥4 (Prediction)			Brier score	ROC area	Observed proportion of dil_cm ≥ 4	Predictive probability of dil_cm ≥4 (prediction)			Brier score	ROC area
		Mean (standard deviation)	Q1-Q3	Min-Max				Mean (standard deviation)	Q1-Q3	Min-Max		
Uterine Activity	0.389	0.389 (0.316)	0.041-0,812	0.041-0.812	0.140	0.865	0.389	0.346 (0.330)	0.041-0.812	0.041-0.812	0.097	0.927
Maternal signs	0.389	0.389 (0.268)	0.135-0,585	0.017-0.844	0.166	0.822	0.389	0.316 (0.242)	0.113-0.462	0.017-0.844	0.145	0.875
Final	0.389	0.389 (0.341)	0.056-0,709	0.004-0.972	0.121	0.905	0.389	0.334 (0.350)	0.021-0.726	0.007-0.963	0.092	0.956

**Fig. 3:**
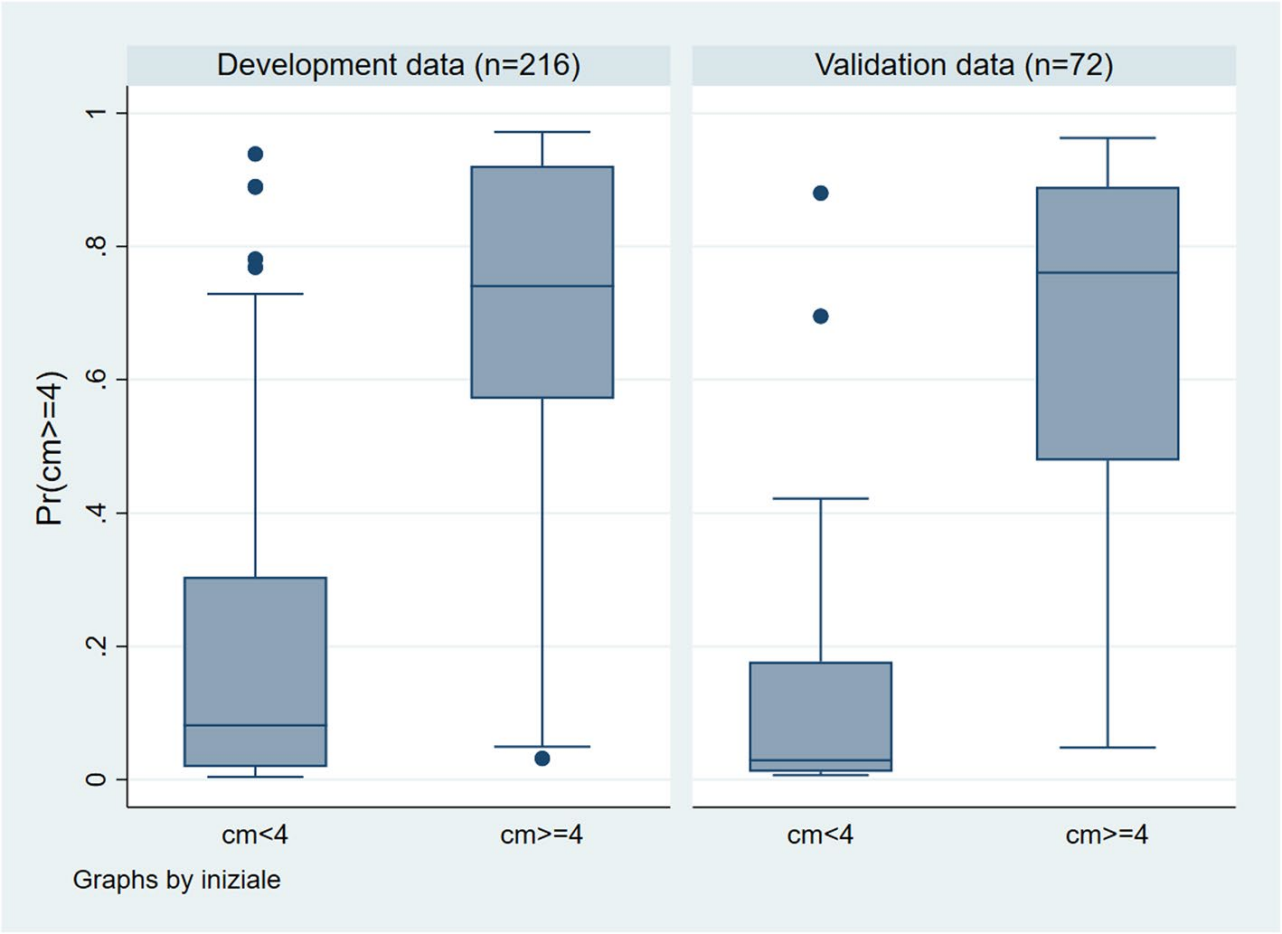
Box plot. The predicted probability of cervical dilatation ≥4 cm is represented in the two groups defined by the observed dilatation (≥4 cm or < 4 cm) in development and validation data

**Fig. 4:**
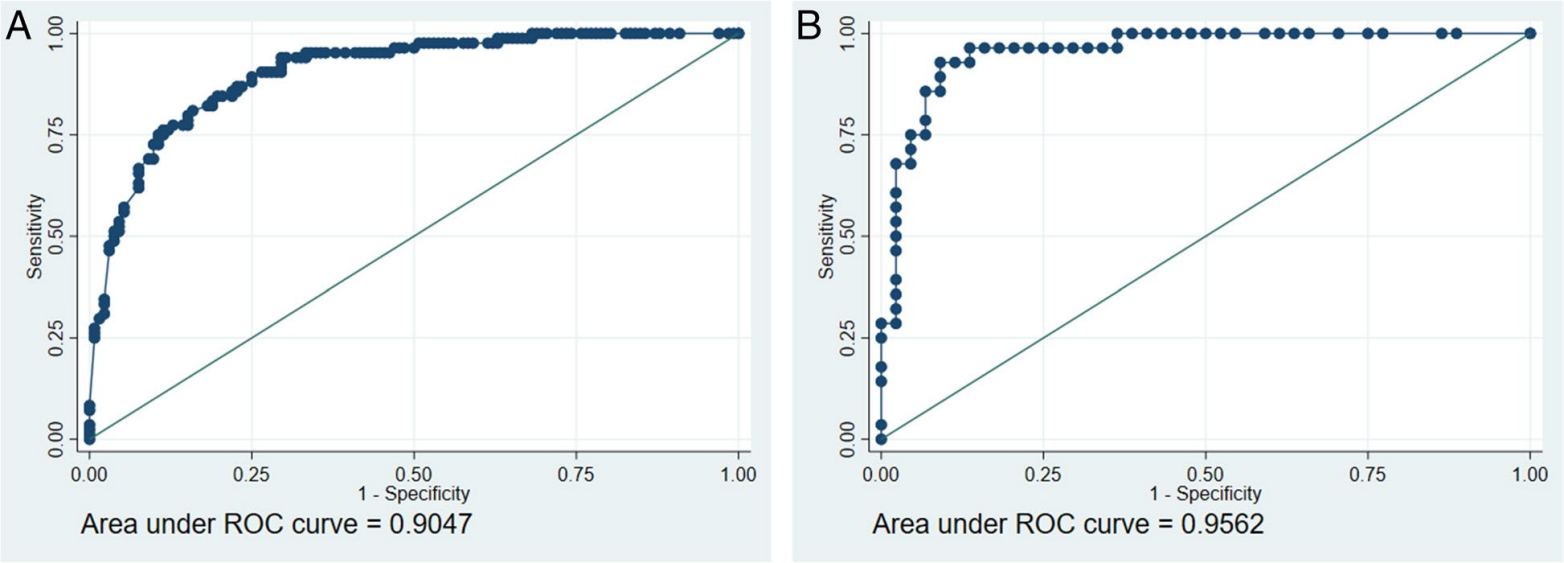
ROC curve. The ROC curve of the predicted probability of cervical dilatation ≥4 cm is represented in development (Panel A) and validation (Panel B) data

The boxplots regarding the prediction of the Final model show a good degree of separation (Fig. 3) confirmed by the ROC analysis (Fig. 4 Panel_A; Panel_B). Again, a slightly better performance is observed in the validation data compared to the development data. The value 0.9047 of the area under the ROC curve of the model predictor applied on the development data (panel A) is the probability that given a pair of women, where one with cervical dilatation ≥4 and one with a cervical dilatation < 4 cm, former had a greater model prediction of being over 4 cm than the latter. This area is 0.9562 when the model predictor is applied on the validation data (panel B).

The analysis of classification errors reported in Tables 6 and 7 enables us to understand the consequences of choosing a fixed threshold to perform the classification. If we consider as an example a threshold equal to 0.4 (a value closed to the observed proportion of assessments with a cervical dilatation ≥4 cm) the probability of having a cervical dilatation ≥4 cm with a prediction lower than the threshold, is 12.3%. While the probability of having a cervical dilatation < 4 cm with a prediction greater or equal than the threshold, is 26.6%. When considering the validation sample, those probabilities are lower.

**Table 6:** Analysis of classification errors when a predictive probability below the threshold suggests a dil_cm < 4

predictive probability threshold	n of assessment below the threshold	% of assessment below the threshold	n of assessment ≥4 (errors)	% of assessment ≥4 (errors)
0.8	184	85.19	55	29.89
0.7	161	74.54	36	22.36
0.6	141	65.28	23	16.31
0.5	134	62.04	19	14.18
0.4	122	56.48	15	12.30
0.3	105	48.61	8	7.62
0.2	91	42.13	4	4.40

**Table 7:** Analysis of classification errors when a predictive probability over the threshold suggests a dil_cm > =4

predictive probability threshold	n of assessment over the threshold	% of assessment over the threshold	n of assessment < 4 (errors)	% of assessment < 4 (errors)
0.8	32	14.81	3	9.38
0.7	55	25.46	7	12.73
0.6	75	34.72	14	18.67
0.5	82	37.96	17	20.73
0.4	94	43.52	25	26.60
0.3	111	51.39	35	31.53
0.2	125	57.87	45	36.00

## Discussion

This study developed a predictive model for the active phase of labor based on maternal signs and symptoms. To our knowledge this is the first study attempting to develop a score for the diagnosis of the active phase of labor. A tool to diagnose the active phase of labor using a non-invasive approach might improve midwives’ skills and assist in the decision making-process, thus having a significant impact on midwifery care. This would avoid unnecessary intrapartum interventions, promoting the normal process of labor [7, 9, 10, 12, 20].

The variables analyzed to develop the score enable us to identify which maternal signs related the most to the active phase of labor.

Uterine contractions have the greatest impact on the diagnosis of active labor. The literature suggested evaluating this variable as a determining factor of labor progress [1, 3, 7, 11, 17, 21]. Women themselves consider uterine contractile activity as the beginning of the active labor in 60% of cases [22]. This is confirmed by the large majority of the studies that attempted to define the active phase of labor [1–3, 6].

The current study adds value to the existing literature because it identified the characteristics of the contractions that are more likely to be associated with a cervical dilatation higher than 4 cm: frequency > 2 contractions in 10 minutes; regular rhythm; length > 50“, highlighting the importance of an accurate midwifery assessment. The final model, which considered features of contractions together with maternal signs, showed that only regular rhythm and duration >50” continued to be significantly associated with a diagnosis of cervical dilatation greater than 4 cm. According to the literature, regular rhythm is the characteristic of contractions strongly associated with the active phase, rather than the latent phase of the first stage of labor [16–18].

All other maternal signs considered in the logistic regression model, were significantly associated with the diagnosis of cervical dilatation greater than 4 cm, highlighting their role in the initial midwifery assessment. However, the final regression model reported that only “show” had a significant impact. The presence of a show is a direct sign of changes on the cervix in terms of effacement and dilation, and this may explain its predictive role. It should be noted that the most significant labor signs, are the ones that have a direct effect on the cervical changes. Other “indirect” signs, more related to changes in the maternal behavior, seem less relevant for the diagnosis of the active phase of labor. Perhaps these signs are more subjective and variable from woman to woman leading to a stronger identification.

The final model had the best performance in both the development and the validation data, suggesting the need to include all factors (features of uterine contractions and maternal signs) within the assessment tool to identify established labor. Our results encourage the evaluation of signs and symptoms to avoid unnecessary vaginal examinations [1, 3, 14, 21].

Furthermore, the inclusion of maternal signs in addition to the uterine activity within the score, reflects the relevance given to the inter-individual variability of woman, who adapts differently to the labor process. Labor is in fact a continuum, a succession of changes within the body and psyche of the woman [17, 23–25]. Only the integration of all components - physical, emotional and behavioral - gives to the midwife a holistic view of the woman and, consequently, more information about the progression of her labor [15–17].

The strengths of the study comprehend the validation of a user-friendly nomogram tool for the diagnosis of active labor, which could be used to facilitate a clinical decision which is too often inaccurate, highly subjective and operator dependent. Despite the small number of women, we have shown that the model is feasible and reliable when applied to the validation data set. Limitations of this study include that the same midwife performed the evaluation of variables included into the assessment tool and the vaginal examination. In addition, we measured the cervical dilatation with a vaginal exam that is an extremely subjective procedure. Furthermore, a number of potentially eligible women were excluded for criteria such as the lack of opportunity of training of midwives or logistic issue that could be avoided in a different organizational condition.

## Conclusions

This study developed a predictive model for the onset of the active phase of labor based on maternal signs and symptoms. The predictive performance of the model on the diagnosis of cervical dilatation more than 4 cm suggests its implementation into clinical practice.

A validated tool based on maternal signs and symptoms would facilitate the initial midwifery assessment process and improve midwives’ confidence and skills, thus embracing a holistic approach to diagnose the onset of the active phase of labor, with the ultimate aim of minimizing interventions. In particular, the tool might be used as a supportive guide to differentiate women who have a valid reason to be assessed through a vaginal examination from the ones who would beneficiate to be discharged home or of a further observation.

In a continuity model of care context, the implementation of this tool might represent a way to achieve a safe and a high quality midwifery care, supporting midwives in the decision making process.

The analysis of classification errors is useful to identify appropriate predictive probability thresholds based on different settings of care and women’s characteristics, adapting the assessment tool to different management of midwifery care.

Further research are warranted to investigate how this tool might associate with maternal and neonatal outcomes, including women’s birth experience.

Moreover, the predictive performance of the assessment tool should be evaluated in different settings and in specific women’s subgroups.

## Data Availability

The datasets generated and/or analyzed during the current study are not publicly available due to the scope of the consent obtained from study participants restricting our ability to share the data on ethical and legal grounds but NMS data are available from the corresponding author on reasonable request.
